# Physician support for diabetes patients and clinical outcomes

**DOI:** 10.1186/1471-2458-9-367

**Published:** 2009-09-29

**Authors:** Jochen Gensichen, Michael Von Korff, Carolyn M Rutter, Michelle D Seelig, Evette J Ludman, Elizabeth HB Lin, Paul Ciechanowski, Bessie A Young, Edward H Wagner, Wayne J Katon

**Affiliations:** 1 Institute for General Practice, University Hospital Jena, Jena, Germany; 2 Group Health Center for Health Studies, Group Health Cooperative, Seattle, WA, USA; 3 Department of Psychiatry and Behavioural Science, University of Washington School of Medicine, Seattle, WA, USA; 4 Veterans Affairs Puget Sound Health System, Seattle, WA, USA

## Abstract

**Background:**

Physician practical support (e.g. setting goals, pro-active follow-up) and communicative support (e.g., empathic listening, eliciting preferences) have been hypothesized to influence diabetes outcomes.

**Methods:**

In a prospective observational study, patients rated physician communicative and practical support using a modified Health Care Climate Questionnaire. We assessed whether physicians' characteristic level of practical and communicative support (mean across patients) and each patients' deviation from their physician's mean level of support was associated with glycemic control outcomes. Glycosylated haemoglobin (HbA1c) levels were measured at baseline and at follow-up, about 2 years after baseline.

**Results:**

We analysed 3897 patients with diabetes treated in nine primary care clinics by 106 physicians in an integrated health plan in Western Washington, USA. Physicians' average level of practical support (based on patient ratings of their provider) was associated with significantly lower HbA1c at follow-up, controlling for baseline HbA1c (*p *= .0401). The percentage of patients with "optimal" and "poor" glycemic control differed significantly across different levels of practical support at follow (*p *= .022 and *p *= .028). Communicative support was not associated with differences in HbA1c at follow-up.

**Conclusion:**

This observational study suggests that, in community practice settings, physician differences in practical support may influence glycemic control outcomes among patients with diabetes.

## Background

Diabetes affects 21 million Americans at an annual cost of over 130 billion dollars. Despite improvements in quality of diabetes care over the last decade, considerable room for improvement remains [1]. The Institute of Medicine has called for "patient-centered" approaches to care, particularly for patients with major chronic conditions such as diabetes [2]. A key question is how primary care physicians can achieve patient-centered care in ways that improved clinical outcomes. In this paper, we assessed two approaches to this end: "communicative support" and "practical support".

Physicians can empower patients by providing choices, being responsive to patient preferences, and understanding, listening, and encouraging patients to ask questions. An approach that affirms the patient's capacity to identify and learn to solve their own problems relies on a patient-centered consultation style and effective communication between doctor and patient [3]. In the last decade, many primary care physicians have been trained to employ these techniques in patient care. However, evidence across studies is inconsistent regarding whether doctor-patient communication that promotes patient autonomy and self-management can, by itself, improve clinical outcomes [4,5].

A complementary view is that improving clinical outcomes depends on practical support from health care teams that facilitate patients' self-management through tangible actions such as: issuing a written care plan agreed on with the patient; setting treatment goals; and providing proactive follow-up to monitor disease control and treatment adherence [6]. The Chronic Care Model advocates "productive interaction" of an "activated patient" with a "proactive clinic team" as a means of improving the clinical outcomes of patients with diabetes and other chronic diseases, placing at least as much emphasis on practical support as on communication [7]. DiMatteo, in a review of interventions to enhance medication adherence, concluded that practical support had greater effects than emotional support [8]. Others have concluded that behavioral interventions focusing directly on patients' behavior are more effective at improving clinical outcomes in diabetes care than changing how physicians communicate with patients [9].

Reviews of controlled trials of diabetes care have concluded that practical support has beneficial effects on clinical outcomes [10]. However, it is unclear how much the type and level of support that primary care teams currently offer affects diabetes clinical outcomes under community practice conditions. In a recent paper in *JAMA*, Pogach et al. observed that: "Although efficacy trials are sufficient for guideline recommendations [...] effectiveness studies, technical considerations (bias, variability in practice and definition of population at risk) [...] are also pertinent" to assess the impact of physician performance on population health among patients with diabetes [11].

This prospective, observational evaluation of the influence of primary care physician support on diabetes outcomes addresses these issues. Specifically, this paper assesses, under community practice conditions, the extent to which two forms of support for patients with diabetes influence glycemic control outcomes. We compare physicians whose patients rate them more (or less) favorably on: a) practical support for diabetes care (e.g., proactive follow-up, setting agreed-on goals, and developing a written action plan); and b) communicative support (e.g., empathic listening and encouraging patients to ask questions). We prospectively assessed the effects of physician support, as evaluated by patients, on glycemic control.

## Methods

### Setting and Patient Sample

A survey was sent to patients with diabetes from nine primary care clinics of Group Health Cooperative, a non-profit integrated health plan in Washington State. Inclusion criteria were ascertained using data from Group Health's diabetes register. Eligibility criteria included: a) taking any anti-diabetic agent; b) fasting glucose ≥126 mg/dl, confirmed by a second test within the next year; c) random plasma glucose ≥200 mg/dl confirmed by a second test within the next year; or d) hospital discharge diagnosis of diabetes or two outpatient diagnoses of diabetes. Further details on subject recruitment for this study are available in a prior report [12]. The study was approved by Group Health's Institutional Review Board.

### Measures of Physician Support

To assess patient perceptions of physician support for diabetes care, we used a modified version of the Health Care Climate Questionnaire (HCCQ) [13]. The original HCCQ assesses physician communicative support for patients' motivation to change health behavior. In the 12-item version we employed [14], HCCQ items assessing communicative support were: 1) I feel my doctor has provided me with choices; 2) I feel understood by my doctor; 3) My doctor conveys confidence in my ability to make changes; 4) My doctor encourages me to ask questions; 5) My doctor listens to what I think; and 6) My doctor tries to understand my view before suggesting a new way to do things. We then augmented the HCCQ with six items assessing practical support for diabetes self-management. These additional items assessed whether the care team: 7) Regularly reviews how patients are doing in managing all aspects of their diabetes; 8) Makes phone calls to find out how patients are doing managing their diabetes; 9) Works with the patients to develop a plan so they know how to take care of their diabetes; 10) Provides a written care plan; 11) Sets personal goals with the patient; and 12) How often the care team makes unsolicited phone calls to check up on the patient. Questions 1 to 9 were scored on a seven-point Likert scale: "strongly disagree" [= 1] to "strongly agree" [= 7]. Questions 10 and 11 were scored "yes" [= 7] or "no or skipped" [= 1]. Question 12 was weighted to the same seven-point scale as the other items: "never" [= 1], "rarely" [= 2.5], "sometimes" [= 4], "often" [= 5.5], and "very often" [= 7].

The modified scale's reliability (Cronbach's Alpha) was high (.87), comparable to the reliability of the original and modified scales. We did an exploratory factor analysis (EFA) with an oblique rotation to identify factors in the scale. Since we expected communicative support and practical support to be correlated, an oblique rotation, which allows the latent variables to be correlated, is appropriate [15]. The EFA identified two major factors (eigenvalue 6.11 and 1.66). A third factor was marginal (eigenvalue 1.05), and only one item loaded on this factor. The oblique rotation showed that items 1 to 6 had high loadings (= 0.4) exclusively on the first factor, while items 8 to 12 had high loadings exclusively on the second factor. Item 7 had high loadings on both factors (Table 1). We refer to these two factors as "communicative support" and "practical support". As expected, the two factors were positively correlated with each other (*r *= .38).

**Table 1:** Factor loadings (oblique rotation) for modified HCCQ Questionnaire

**Item**	**Origin**	**Factor 1**	**Factor 2**
1) I feel my doctor has provided me with choices	HCCQ	0.73755	0.16272
2) I feel understood by my doctor	HCCQ	0.83308	0.10529
3) My doctor conveys confidence in my ability to make changes	HCCQ	0.81818	0.06163
4) My doctor encourages me to ask questions	HCCQ	0.86087	0.05970
5) My doctor listens to what I think	HCCQ	0.89648	0.01723
6) My doctor tries to understand my view before suggesting a new way to do things.	HCCQ	0.86835	0.05433
7) My doctor regularly reviews with me how I am doing in managing all aspects of my diabetes	Supplement	0.44656	0.52897
8) My doctor makes calls to find out how I am doing managing my diabetes	Supplement	0.06431	0.77974
9) My doctor have worked with me to develop a plan so I know how to take care of my diabetes	Supplement	0.33954	0.62477
10) Do you have a copy of the plan in writing	Supplement	-0.05039	0.52797
11) Do you work with your doctor to set sets personal goals	Supplement	0.06520	0.67932
12) How often did the doctor call to check and see how you were doing without you calling him first.	supplement	-0.11627	0.78835

We evaluated the association of the scales with patient self-care behavior such as patient's diet, exercise, and glucose monitoring (Diabetes Self Care Scale) [16]. Frequency of glucose monitoring was significantly correlated with the practical support subscale (*r *= .18) and the communicative support subscale (*r *= .08). Practical and communicative support were also significantly correlated with patients' depression levels as assessed by the Patient Health Questionnaire (PHQ-9) [17], (*r *= -.20 for communicative support and *r *= -.16 for practical support). Correlations were modest suggesting that these were not simply measuring patients' global positive or negative attitudes.

### Measures of Glycemic Control

We obtained glycosylated hemoglobin (HbA1c) levels at baseline and follow-up from Group Health electronic medical records. The baseline HbA1c assessment was the first test identified at least three months (but no more than 24 months) before date of study assessment. The follow-up HbA1c was the first test occurring at least three months (but no more than 36 months) after the date of study assessment. The average of the interval between HbA1c readings was 23 months, providing an extended time period to observe effects of communicative and practical support on glycemic control.

### Analysis

Using automated data on survey respondents and survey non-respondents, we were able to estimate response propensity scores (the probability of being a respondent) as a function of clinical and socio-demographic variables [18] We predicted response/non-response status as a function of these variables using logistic regression as implemented by PROC LOGISTIC of SAS. Using the predictors, we estimated a response probability for each survey respondent (the response propensity score). We divided this response probability into one to estimate a response probability adjusted analysis weight for each respondent. In a weighted analysis, persons with a low probability of responding would be given a higher weight in the analysis to represent the larger number of non-respondents with similar characteristics.

We used linear regression to estimate the association between baseline support measures and HbA1c levels at follow-up. Regression models adjusted for patient characteristics known or expected to be related to HbA1c at follow-up: baseline HbA1c, baseline PHQ-9; age, sex, educational level, duration of diabetes, and insulin use status (any use vs. none) and propensity score for non-response. Since HbA1c may change with time (e.g. aging effects), we also controlled for the number of months between the baseline and follow-up HbA1c measurement for each patient. We estimated the regression model using generalized estimating equations (GEE) [19] with an exchangeable working correlation matrix to account for clustering of patients within physicians.

Since measures of practical support vary both across physicians and between patients seen by the same physician, we used an approach developed by Neuhaus [20] to differentiate between-physician and within-physician covariate effects. Between-physician effects capture systematic differences between providers that are attributable to the average perceived support reported by their patients. These are estimated by including the mean support reported by their patients. Within-physician effects capture patient-level effects of perceived support, and are estimated by each patient's deviation from their physician's mean level of support. That is, between- and within-physician effects differentiate effects that occur at the physician level from those that occur on the patient level. Models that do not explicitly distinguish within- from between-physician effects estimate a mixture of both.

The regression models include four measures of support: the physician's mean level of practical support and communicative support, and each patient's deviation from the physician mean for communicative and practical support.

Pogach et al. recommended population-based measures of glycemic control that convey the extent of "poor" (HbA1c >9%) and "optimal" (HbA1c <7%) glycemic control [11], in addition to reporting mean glycemic control. In line with this recommendation, we carried out analyses of the percentage of patients with "optimal" and "poor" control for physicians who provided low (<20%), medium (20-80%), or high (>80%) levels of scores in practical and communicative support.

## Results

The questionnaire was sent to 9063 patients from the Group Health registry between March 2001 and August 2002, of whom 7841 were eligible for the study. Questionnaires were returned by 4839 (61.7%). We included 3897 patients from 106 physicians. The number of patients per physician ranged from 5 to 105 with a mean of 40.3 patients (*SD *24.1) per physician. We excluded patients who: did not give their consent to a review of automated medical records (n = 369); did not have two HbA1c tests available at least six months apart (*n *= 365); did not have valid data for the Health Care Climate Questionnaire (*n *= 165); did not report their educational level (*n *= 39); and/or whose primary care physician had fewer than five patients with diabetes included in the study (*n *= 4 patients). Almost half were older than 65 years (47.8%), 20.1% were non-Caucasian, about two thirds had not graduated from college, and 95.6% had a type II diabetes. At baseline, 73.1% had an HbA1c >7%, and 24.1% had no oral hypoglycemic or insulin treatment (Table 2).

**Table 2:** Baseline characteristics of patients (N = 3897)

**Variable**	**Percentage of patients (%)**
**Age (years)**	
<65	52.1
**Sex**	
Female	48.3
**Race**	
White/Caucasian	79.9
Black/African American	8.2
Asian or Pacific Islander	9.0
American Indian/Alaskan Native	1.5
Other	1.4
**Education**	
<College graduate	64.6
≥College graduate	35.3
**Marital Status**	
Unmarried	33.0
Married	66.9
**Employment**	
No	56.7
Yes	43.2
**HbA_**1c **_baseline**	
<7.0% "optimal" control	26.8
>9.0% "poor" control	25.1
**Treatment intensity**	
No treatment	24.1
Oral hypoglycemic only	46.2
Insulin or/and oral hypoglycemic	29.6

The mean time interval between baseline and follow-up HbA1c measurement was 23.1 (SD 4.4) months, with minimum and maximum intervals of 7.4 and 55 months, respectively. The mean HbA1c value was 8.1% at baseline and 7.5% at follow-up, reflecting concurrent national trends toward improvement in glycemic control [1].

The prospective analysis of predictors of HbA1c at follow-up found that being seen by a physician with a higher mean level of practical support was associated with more favorable glycemic control outcomes. Baseline HbA1c, the other indicators of diabetes severity, and the time interval between baseline and follow-up HbA1c measures were also significant predictors of follow-up HbA1c (Table 3). After controlling for the case-mix variables, the physician's mean level of practical support was a significant predictor of follow-up HbA1c (*p *= .040). The patient-level deviation from the physician's mean level of practical support was not associated with differences in HbA1c outcomes. In contrast, neither the physician's mean level of communicative support, nor the patient deviation from the physician's mean, was associated with differences in HbA1c outcomes.

**Table 3:** Linear regression model (GEE) for weighted associations with follow-up HbA1c

**Parameter**	**Estimate**	**95% Confidence interval (*CI*)**	***P *value**
Years with diabetes	0.0123	0.0066 - 0.0179	<.0001
Insulin use	0.2004	0.0717 - 0.3291	<.0001
Months between HbA_1c _measures	0.0463	0.02307 - 0.0619	<.0001
Baseline HbA_1c_	0.3857	0.3524 - 0.4246	<.0001
Baseline PHQ-9	0.0077	-0.0008 - 0.0161	.0753
			
Practical support (physician mean)	-0.1787	-0.3494 - -0.0081	.0401
Practical support (individual patient as deviation from physician mean)	0.0196	-0.0158 - 0.0550	.2779
Communicative support (physician mean)	0.1053	-0.0689 - 0.2796	.3361
Communicative support (individual patient as deviation from physician mean)	0.0067	-0.0381 - 0.0514	.7702

Analysis of GEE Parameter Estimates Empirical Standard Error Estimates; Correlation = .0108; adjusted for age, sex, and education, propensity score for non-respondent

Table 4 shows the proportion of patients with "poor" and "optimal" HbA1c by the average level of practical support and communicative support that the clinic team provided at baseline and at follow-up. The top data compares the percentage of patients with "optimal" control (HbA1c <7%). At baseline, differences were not significant, whereas at follow up the percentage with "optimal" control differed significantly across the three levels of practical support. Likewise, the percentage with "poor" control (HbA1c >9%) was somewhat (but not significantly) lower in the low-support group at baseline, but the percentage with "poor" control differed significantly at follow-up across the three levels of practical support (at the bottom). It is important to note that the differences in trends in the percentage with "optimal" and "poor" glycemic control were modest across the three levels of communicative and practical support. However, the observed differences in glycemic control trends emerged over a follow-up period averaging 23 months.

**Table 4:** Proportion of patients with unadjusted HbA1c by average level of communicative and practical support that clinic team provided at baseline and at follow-up (mean, 24 months).

**Optimal glycemic control (HbA_**1c **_<7%)**								
	**Communicative support**				**Practical support**			
	**Low**	**Medium**	**High**	***P***	**Low**	**Medium**	**High**	***P***

**Baseline** **N = 1045**	28.7%	26.3%	26.1%	**n.s.**	23.8%	27.7%	26.4%	**n.s.**
**Follow-up** **N = 1518**	40.6%	39.0%	35.5%	**n.s.**	34.5%	39.6%	40.9%	**.022**

**Poor glycemic control (HbA_**1c **_>9%)**								

	**Communicative support**				**Practical support**			
	**Low**	**Medium**	**High**	***P***	**Low**	**Medium**	**High**	***P***

**Baseline** **N = 1042**	24.0%	26.9%	30.1%	**n.s.**	28.6%	26.6%	24.9%	**n.s.**
**Follow-up** **N = 583**	14.2%	14.7%	17.4%	**n.s.**	17.6%	14.8%	12.6%	**.028**

Percentage of patients with "optimal" and "poor" control for physicians who provided low (<20%), medium (20-80%), or high (>80%) levels of scores in providers' practical and communicative support

## Discussion

In response to a call for community-based research on how clinical care influences diabetes outcomes [11], this paper employed an observational, prospective design to assess implications of differences in practical support and communicative support from primary care physicians on glycemic control outcomes among patients with diabetes. The key result of this study was that physicians' characteristic level of practical support (as rated by their patients) was associated with more favorable glycemic control outcomes after controlling for baseline HbA1c and other measures of diabetes severity. Contrary to expectation, physicians' level of communicative support was not associated with differences in glycemic control outcomes, although practical and communicative support were correlated.

Since this study was observational, these results need to be considered in light of relevant experimental studies. The results of this observational study are generally consistent with findings from prior experimental and observational studies concerning effects of clinic team support for diabetes self-management. A growing body of experimental research has shown beneficial effects on clinical outcomes of interventions that enhance the organization of diabetes care in ways that afford practical support for patient self-management [10]. This observational research extends the experimental results by suggesting that differences in practical support observed in community practice settings may influence long-term trends in glycemic control. In contrast, interventions that have targeted the doctor-patient communication style in diabetes care have not consistently shown effects on patient outcomes. In controlled studies, physician training improved physicians' communication skills and patients' well-being and satisfaction - but not patients' HbA1c levels [21].

Schillinger trained physicians in communications skills and found positive effects on HbA1c (<8.6%) for patients with low health literacy [22]. These inconsistent results concerning benefits of enhanced doctor-patient communication have also been reported for other chronic conditions. While experimental studies with direct observation of doctor-patient communication have shown that it is possible to improve the quality of communication, evidence is limited that such changes improve clinical outcomes [23]. A recent review reported improved clinical outcome in only two of seven provider-targeted interventions to improve patient participation [24]. In Griffin's review, almost half of the 11 studies focused on provider communication skills training programs found at least one worsened outcome in the intervention group [4]. The contribution of this paper is to suggest that differences in practical support observed across physicians in community practice settings may influence glycemic control among persons with diabetes.

In ambulatory diabetes care in community practice, the primary task is to minimize the incidence of "poor" glycemic control, while maximizing the prevalence of "optimal" control is a secondary goal [11]. Our findings suggest that differences in levels of practical support offered by community-based health care teams may influence glycemic control outcomes over an observation period of about 2 years. In contrast, individual patient differences in practical support levels, from their physician's mean level of practical support, did not predict differences in glycemic control outcomes. Features of practical support - such as proactive follow-up, setting agreed-on goals, and developing a written action plan - require organization of the primary care team. Therefore, a physician's typical level of practical support may reflect important differences not only in physician care, but in how well care is organized by the entire clinic team - including other team members than the physician [25]. It is also possible that using information from multiple patients yielded more reliable and valid measurement of practical support provided by health care teams. It is interesting that in our analysis communicative support for diabetes care alone was not associated with differences in glycemic control outcomes. Patient ratings of communicative support were correlated with their ratings of practical support, so these two forms of support are interrelated. Communicative support may be necessary, but insufficient by itself to affect patient outcomes. On the other hand, practical support may complement doctor-patient communication. The findings of this observational study suggest that an organized approach to providing practical support for self-management influenced long-term trends in glycemic control. The benefits of practical support may be augmented by effective doctor-patient communication and a relationship that empowers patients in managing their disease [26,27].

Limitations of this study need to be considered in interpreting the results. Provider support was determined by ratings of multiple patients rather than by direct observation. Patients' global ratings of health care quality have been shown to be unrelated to objective overall measures of technical quality of care [28]. However, we measured patients' perceptions of specific behaviorally defined aspects of patient care that have been found useful in prior research, particularly when results are aggregated across larger numbers of patients [29]. Due to constraints on questionnaire length, an abbreviated questionnaire was used to assess physicians' practical and communicative support. Ideally, the effects of practical and communicative support would be assessed through experimental manipulation and direct observation of support, but such a study would be expensive and difficult. This observational study sheds light on the implications of differences in support levels observed in community practice rather than support levels achieved under experimental conditions. In contrast to prior observational studies assessing effects of provider support on diabetes outcomes, we adjusted for important confounders, specifically baseline HbA1c and clinical factors associated with HbA1c. Since poor glycemic control may influence the care received by patients with diabetes (more or less attention and support), the use of a prospective design controlling for baseline HbA1c is an important feature of this study. However, it is, of course, possible that other unmeasured confounders may explain differences in glycemic control outcomes between patients managed by physicians rated as providing high versus low levels of practical support.

## Conclusion

This prospective, observational study suggests that physicians who typically offer higher levels of practical support for diabetes self-management, their patients achieve more favorable glycemic control at follow-up. While patient ratings of physicians' communicative support were not associated with glycemic control, communicative and practical support were correlated so these two forms of support may be viewed as complementary. Practical support seems to be a critical part of the doctor-patient relationship and is an effective part of physician support for patient self-management. These results are consistent with prior experimental and observational research, suggesting that differences in practical support for diabetes self-management that occur in community practice settings can make a difference in patient outcomes.

## Competing interests

The authors declare that they have no competing interests.

## Authors' contributions

JG: Conception and design, analysis and interpretation, statistical analysis, drafting of the article, critical revision of the article for important intellectual content, final approval of the article, obtaining funding. MvK: Conception and design, data collection, analysis and interpretation, statistical analysis, drafting of the article, critical revision of the article for important intellectual content, final approval of the article, obtaining funding. CMR: Conception and design, data collection, analysis and interpretation, statistical analysis, drafting of the article, critical revision of the article for important intellectual content, final approval of the article, obtaining funding. MDS: Conception and design, analysis and interpretation, statistical analysis, drafting of the article, critical revision of the article for important intellectual content, final approval of the article, obtaining funding. EJL: Data collection, analysis and interpretation, critical revision of the article for important intellectual content, final approval of the article. EHBL: Data collection, analysis and interpretation, critical revision of the article for important intellectual content, final approval of the article. PC: Data collection, analysis and interpretation, critical revision of the article for important intellectual content, final approval of the article. BAY: Data collection, analysis and interpretation, critical revision of the article for important intellectual content, final approval of the article. EHW: Analysis and interpretation, critical revision of the article for important intellectual content, final approval of the article. WJK: Conception and design, data collection, analysis and interpretation, critical revision of the article for important intellectual content, final approval of the article, obtaining funding.

## Pre-publication history

The pre-publication history for this paper can be accessed here:


